# Exploring *APC* Mosaicism in Upper Intestinal Tract Adenomas

**DOI:** 10.1200/PO-25-00027

**Published:** 2025-09-16

**Authors:** Diantha Terlouw, Maartje Nielsen, Jurjen J. Boonstra, Monique E. van Leerdam, Tom van Wezel, Hans Morreau, Alexandra M.J. Langers

**Affiliations:** ^1^ Department of Pathology, Leiden University Medical Center, Leiden, the Netherlands; ^2^ Department of Clinical Genetics, Leiden University Medical Center, Leiden, the Netherlands; ^3^ Department of Gastroenterology and Hepatology, Leiden University Medical Center, Leiden, the Netherlands

## Introduction

Familial adenomatous polyposis (FAP) is caused by germline pathogenic *APC* variants and is characterized by the development of hundreds to thousands colorectal adenomas.^1^ Moreover, duodenal adenomas are observed in 50%-90% of patients with FAP, increasing the lifetime risk of small bowel carcinomas up to 12%.^2^

*APC* mosaicism accounts for 25%-50% of patients diagnosed with more than 20 colorectal adenomas without a germline predisposition.^3,4^ Whether *APC* mosaicism is associated with the development of multiple adenomas in the upper intestinal tract without the development of multiple colorectal adenomas is still largely unknown. In this report, we describe two cases of isolated duodenal *APC* mosaicism.

## Case Presentation

### Case 1

**FIG 1.**
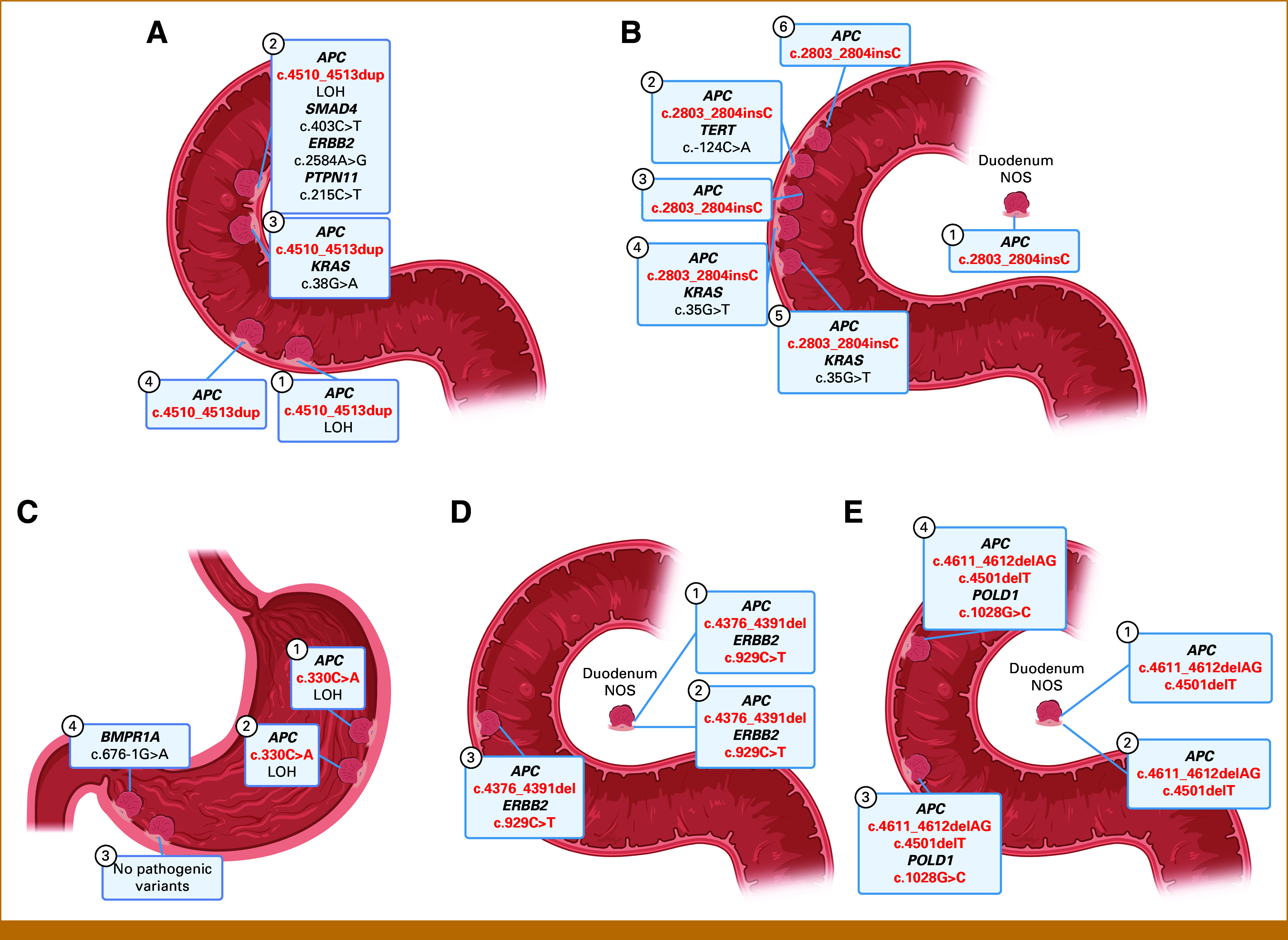
Patients with at least two adenomas sharing the same pathogenic *APC* variant. (A; Patient ID No.: 1) The first duodenal mosaicism case presented in this report with the pathogenic *APC* c.4510_4513dup variant in all duodenal adenomas but not in colorectal adenoma or other tissues tested. (B; Patient ID No.: 2) The second duodenal mosaicism case with the pathogenic *APC* c.2803_2804insC variant in all duodenal adenomas. (C; Patient ID No.: 3) Patient with two gastric adenomas in location T1 and T2 both having *APC* c.330C>A, whereas this variant is absent in T3 and T4, suggesting clonal relationship. A rectum adenoma showed two distinct pathogenic *APC* variants (Data Supplement, Table S1). (D; Patient ID No.: 4) Three duodenal lesions sharing the same pathogenic *APC* and *ERBB2* variant which concludes clonal relationship. (E; Patient ID No.: 5) Patient with four duodenal lesions sharing two pathogenic *APC* variants. T3 and T4 also harbor the same *POLD1* VUS. *POLD1* is technically not covered in T1 and T2. Two colorectal adenomas did not show any of the *APC* or *POLD1* variants (Data Supplement, Table S1). Created using Biorender.com. LOH, loss of heterozygosity; NOS, not otherwise specified; VUS, variant of unknown significance.

The first case is a 61-year-old patient who presented with unspecified upper abdominal pain, underwent an esophagogastroduodenoscopy (EGD), and was diagnosed with an ampullary adenoma with an extensive peripapillary component and more than 10 additional duodenal adenomas in the descending and horizontal part of the duodenum, ranging in size from several millimeters to over 2 cm. No abnormalities were detected in the stomach. Since periampullary adenomas are present in at least 50% of patients with FAP,^5^ the suspicion of FAP was raised. In the colorectum, however, only one nonadvanced rectal adenoma was detected during multiple colonoscopies performed previously, because of a positive paternal family history for colorectal cancer. Targeted next-generation sequencing (NGS) was conducted on four duodenal adenomas and the rectum adenoma using a custom-made “msCRC” panel. The msCRC panel consists of 20 colorectal cancer– and polyposis-associated genes like *APC*, *MUTYH*, *POLE*, *POLD1*, *BMPR1A*, and *TP53*.^6^ Manufacturer's instructions were followed to prepare the NGS libraries, and sequencing was performed using the Ion GeneStudio S5 Series sequencer (Thermo Fisher Scientific, Waltham, MA). Figure 1A shows that the same pathogenic *APC* variant, NM_00038.5:c.4510_4513dup, present is in all tested duodenal adenomas and absent in the rectum adenoma. To exclude a clonal relationship, two of the duodenal adenomas were analyzed using a small custom-made targeted “Cancer Hotspot” panel covering hotspot mutations in oncogenes and tumor suppressor genes. Library preparation and sequencing of this panel were performed on the Genexus Integrated Sequencer (Thermo Fisher Scientific). Cancer Hotspot analysis showed unique somatic pathogenic variants in *SMAD4* and *ERBB2* and a variant of unknown significance (VUS) in *PTPN11* in T2 and in *KRAS* in T3. The presence of an identical *APC* variant in combination with different somatic variants in other genes excludes a clonal relationship and confirms mosaicism. Using “tumor-informed” approach, reaching a detection limit of around 1%, the mosaic *APC* variant was not detectable in leukocyte, urine, and buccal swab DNA. This suggests a mosaicism restricted to the duodenum. A follow-up EGD was planned 1 year after the initial EGD. The interval of next EGDs depends on the findings in previous EGDs and will be based on the European FAP Consortium protocol for upper GI tract surveillance in FAP.^7^ Although the mosaic variant was absent in the one rectal adenoma, a follow-up colonoscopy was advised.

## Case 2

The second case is a 25-year-old patient who presented with unspecified abdominal pain. EGD showed a sliding hiatal hernia, antrum gastritis, and duodenitis with a duodenal tubular adenoma. After the initial EGD, the patient developed five duodenal adenomas and two colorectal hyperplastic polyps in the following 13 years. Given the young age at developing duodenal adenomas, germline diagnostic sequencing of the most common polyposis-associated genes (*APC*, *MUTYH*, *NTHL1*, *POLE*, *POLD1*, *AXIN2*, and *MLH3*) was performed on leukocyte DNA, a “tumor-naïve” approach. This analysis showed a germline pathogenic monoallelic *MUTYH* variant, which is not likely to explain the development of duodenal adenomas.^8^ Subsequently, NGS msCRC was performed on all available duodenal adenomas. As depicted in Figure 1B, a recurrent pathogenic *APC* variant (NM_00038.5:c.2803_2804insC) was detected in all adenomas tested. Additional Cancer Hotspot analyses showed distinct somatic pathogenic variants in *KRAS* and *TERT*. The *APC* variant (c.2803_2804insC) was furthermore detected in normal duodenal mucosa adjacent to a tubular adenoma but not in two colonic hyperplastic polyps, again suggesting *APC* mosaicism restricted to the duodenum. Future follow-up EGDs will be planned according to the European FAP Consortium protocol,^7^ with some modifications after discussing the suggested interval with the patient.

### Upper Intestinal Tract Adenoma Cohort

Based on the findings in these two patients, NGS msCRC was performed on the upper intestinal tract adenomas of 13 additional patients to investigate the presence or the extent of *APC* mosaicism in these patients. This cohort included the following: (1) six patients with multiple upper intestinal adenomas including 3-12 duodenal or gastric adenomas with the first diagnosed at age between 45 and 72 years (patient ID 3-8), (2) three patients with one duodenal adenoma in combination with multiple colorectal adenomas (patient ID 9-11), and (3) four known colorectal *APC* mosaicism patients with upper intestinal adenomas (patient ID 12-15). The reason to include patients with multiple upper GI adenomas (1) and the patients with one duodenal adenoma in combination with multiple colorectal adenomas (2) was to investigate whether the clinical findings in these patients could also be explained by *APC* mosaicism. The reason to include the patients with known colorectal *APC* mosaicism in combination with duodenal adenomas (3) was to investigate whether the duodenal adenomas in these patients could be explained by a manifestation of the *APC* mosaicism in the duodenum as well. Clinical characteristics, genes sequenced in the germline, and somatic sequencing results are summarized in the Data Supplement (Table S1).

**FIG 2.**
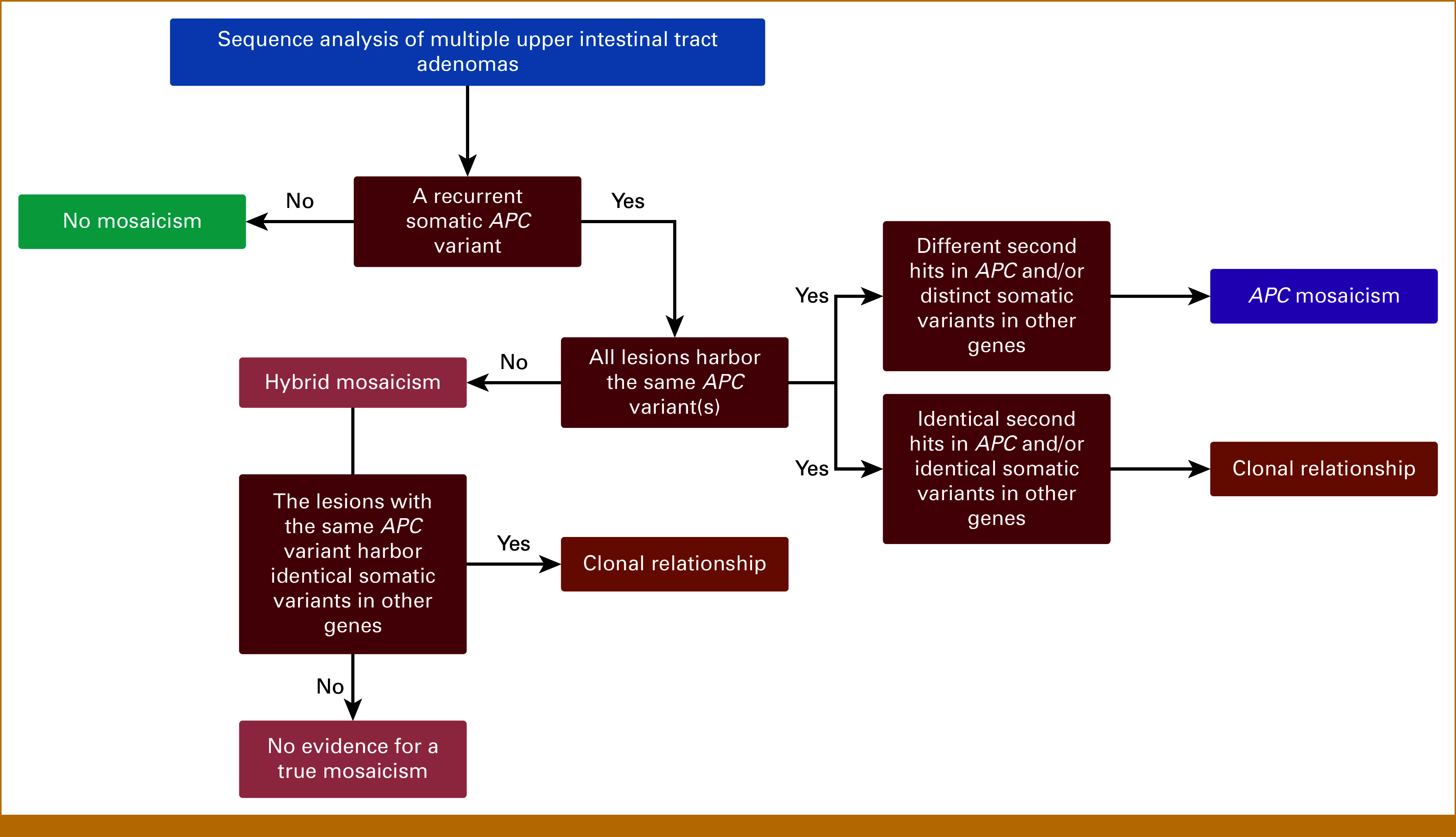
Decision tree for the interpretation of sequencing results of multiple GI tract adenomas.

No *APC* mosaicism was detected in the six patients with multiple upper intestinal adenomas and three patients with one duodenal and multiple colorectal adenomas. However, our results suggest that some of these cases could be explained by either hybrid *APC* mosaicism or clonal relationship. Three patients (ID 3-5) showed identical *APC* variants in at least two of the analyzed gastric/duodenal adenomas, as depicted in Figures 1B and 1D and the Data Supplement (Table S1). In ID 3 (Fig 1C), two of four gastric adenomas (T1 and T2) harbor the same pathogenic *APC* variant, c.330C>A. No other somatic variants were detected. The variant was not detectable in two other gastric adenomas (T3 and T4), and because T1 and T2 were located close to each other, we concluded that there is no convincing evidence for mosaicism and T1 and T2 are probably clonally related. Furthermore, a rectum adenoma showed two distinct *APC* variants. Another patient, ID 4 (Fig 1D), harbored the pathogenic *APC* variant c.4376_4391del and an identical pathogenic *ERBB2* in all duodenal adenomas tested, suggesting a clonal relationship. The third patient, ID 5 (Fig 1E), had two shared pathogenic *APC* variants in four duodenal adenomas, the first and second hit. In T3 and T4, an identical somatic *POLD1* VUS was detected, which was not covered in the sequencing analyses of T1 and T2. Still, the two shared *APC* variants in T1-T4 combined with the *POLD1* variant in T3 and T4, and the fact that the adenomas were removed during four consecutive EGDs suggests a recurrence, so clonal relationship. Two colorectal adenomas in this patient's (recto)sigmoid did not show these *APC* or *POLD1* variants. More background information on the classification and interpretation of the findings of the sequencing analysis can be found in the decision tree in Figure 2.

All duodenal adenomas of four colorectal *APC* mosaicism patients showed the mosaic variant found in colorectal adenomas, as summarized in the Data Supplement (Table S1) and described before.^9^ In one of these patients, the mosaic variant was also detected in leukocyte, urine, and buccal swab DNA, showing extensive mosaicism.

The two patients whose case is described in the manuscript both provided consent to publish their information. Their consent is documented in their electronic patient file.

## Discussion

Although previous studies^9-11^ described upper intestinal tract adenomas in *APC* mosaic patients, to our knowledge, this study is the first to report solitary duodenal *APC* mosaicism and to examine *APC* mosaicism in patients with duodenal adenomas regardless of the presence of colorectal adenomatous polyps. Moreover, this report provides a decision tree for the interpretation of sequencing results of multiple GI tract adenomas, which helps to clarify the differences between the clonal relationship, pure *APC* mosaicism and hybrid *APC* mosaicism.

Future studies with larger cohorts could help provide recommendations on when to test for *APC* mosaicism in patients with upper intestinal tract adenomas. However, given the rarity of the presence of multiple duodenal adenomas, our findings underscore the importance of considering molecular testing for *APC* mosaicism, especially in the case of multiple duodenal or periampullary adenomas.

In addition, more extensive studies should elaborate on the recommendations for clinical management of patients with duodenal *APC* mosaicism. Currently, we would recommend to surveil these patients according to upper GI tract surveillance protocols for patients with FAP as we did in these two cases. Moreover, at least one colonoscopy should be considered to rule out colonic adenomas and a more extensive mosaicism. The interval for follow-up colonoscopies should be determined based on the findings of the index colonoscopy. As there is currently no possibility to completely rule out the presence of colorectal *APC* mosaicism in patients diagnosed with duodenal *APC* mosaicism, we would advise repeating the colonoscopy after 10 years in patients younger than 50 years, in whom no colorectal polyps were identified during their initial colonoscopy.

Vice versa, our study emphasizes the risk of developing upper intestinal tract adenomas in colorectal *APC* mosaicism cases, highlighting the relevance of advising at least one gastroduodenoscopy.

## References

[1] Kinzler KW, Vogelstein B: Lessons from hereditary colorectal cancer. Cell 87:159-170, 1996 8861899 10.1016/s0092-8674(00)81333-1

[2] Kadmon M, Tandara A, Herfarth C: Duodenal adenomatosis in familial adenomatous polyposis coli. A review of the literature and results from the Heidelberg Polyposis Register. Int J Colorectal Dis 16:63-75, 2001 11355321 10.1007/s003840100290

[3] Jansen AML, Crobach S, Geurts-Giele WRR, et al: Distinct patterns of somatic mosaicism in the APC gene in neoplasms from patients with unexplained adenomatous polyposis. Gastroenterology 152:546-549.e3, 2017 27816598 10.1053/j.gastro.2016.10.040

[4] Spier I, Drichel D, Kerick M, et al: Low-level APC mutational mosaicism is the underlying cause in a substantial fraction of unexplained colorectal adenomatous polyposis cases. J Med Genet 53:172-179, 2016 26613750 10.1136/jmedgenet-2015-103468

[5] Mehta NA, Shah RS, Yoon J, et al: Risks, benefits, and effects on management for biopsy of the papilla in patients with familial adenomatous polyposis. Clin Gastroenterol Hepatol 19:760-767, 2021 32492482 10.1016/j.cgh.2020.05.054

[6] Terlouw D, Suerink M, Boot A, et al: Recurrent APC splice variant c.835-8A>G in patients with unexplained colorectal polyposis fulfilling the colibactin mutational signature. Gastroenterology 159:1612-1614.e5, 2020 32603656 10.1053/j.gastro.2020.06.055

[7] Aelvoet AS, Pellise M, Bastiaansen BAJ, et al: Personalized endoscopic surveillance and intervention protocols for patients with familial adenomatous polyposis: The European FAP Consortium strategy. Endosc Int Open 11:E386-E393, 2023 37102182 10.1055/a-2011-1933 PMC10125778

[8] El Hachem N, Abadie C, Longy M, et al: Endoscopic phenotype of monoallelic carriers of MUTYH gene mutations in the family of polyposis patients: A prospective study. Dis Colon Rectum 62:470-475, 2019 30640315 10.1097/DCR.0000000000001323

[9] Terlouw D, Hes FJ, Suerink M, et al: APC mosaicism, not always isolated: Two first-degree relatives with apparently distinct APC mosaicism. Gut 72:2186-2187, 2023 36307181 10.1136/gutjnl-2022-328540

[10] Ciavarella M, Miccoli S, Prossomariti A, et al: Somatic APC mosaicism and oligogenic inheritance in genetically unsolved colorectal adenomatous polyposis patients. Eur J Hum Genet 26:387-395, 2018 29367705 10.1038/s41431-017-0086-y PMC5839046

[11] Takao M, Yamaguchi T, Eguchi H, et al: APC germline variant analysis in the adenomatous polyposis phenotype in Japanese patients. Int J Clin Oncol 26:1661-1670, 2021 34106356 10.1007/s10147-021-01946-4

